# Lipoprotein(a) and risk-weighted apolipoprotein B: a novel metric for atherogenic risk

**DOI:** 10.1186/s12944-024-02307-6

**Published:** 2024-09-27

**Authors:** Michaela B. Rehman, Benoit V. Tudrej

**Affiliations:** 1 Cardiology Department, Ramsay Santé, Médipôle Lyon-Villeurbanne, 158 rue Léon Blum, Villeurbanne, 69100 France; 2 Université Claude Bernard Lyon 1, University College of General Medicine, 8 Avenue Rockefeller, Lyon, 69008 France

**Keywords:** Lipoprotein(a), Apolipoprotein B, Atherosclerotic cardiovascular disease, Coronary heart disease, Risk assessment, Novel metric

## Abstract

**Background:**

Retention of apolipoprotein B (apoB)-containing lipoproteins within the arterial wall plays a major causal role in atherosclerotic cardiovascular disease (ASCVD). There is a single apoB molecule in all apoB-containing lipoproteins. Therefore, quantitation of apoB directly estimates the number of atherogenic particles in plasma. ApoB is the preferred measurement to refine the estimate of ASCVD risk. Low-density lipoprotein (LDL) particles are by far the most abundant apoB-containing particles. In patients with elevated lipoprotein(a) (Lp(a)), apoB may considerably underestimate risk because Mendelian randomization studies have shown that the atherogenicity of Lp(a) is approximately 7-fold greater than that of LDL on a per apoB particle basis. In subjects with increased Lp(a), the association between LDL-cholesterol and incident CHD (coronary heart disease) is increased, but the association between apoB and incident CHD is diminished or even lost. Thus, there is a need to understand the mechanisms of Lp(a), LDL-cholesterol and apoB-related CHD risk and to provide clinicians with a simple practical tool to address these complex and variable relationships. How can we understand a patient’s overall lipid-driven atherogenic risk? What proportion of this risk does apoB capture? What proportion of this risk do Lp(a) particles carry? To answer these questions, we created a novel metric of atherogenic risk: risk-weighted apolipoprotein B.

**Methods:**

In nmol/L: Risk-weighted apoB = apoB - Lp(a) + Lp(a) x 7 = apoB + Lp(a) x 6. Proportion of risk captured by apoB = apoB divided by risk-weighted apoB. Proportion of risk carried by Lp(a) = Lp(a) × 7 divided by risk-weighted apoB.

**Results:**

Risk-weighted apoB agrees with risk estimation from large epidemiological studies and from several Mendelian randomization studies.

**Conclusions:**

ApoB considerably underestimates risk in individuals with high Lp(a) levels. The association between apoB and incident CHD is diminished or even lost. These phenomena can be overcome and explained by risk-weighted apoB.

## Background

Atherosclerotic cardiovascular disease (ASCVD) is a leading cause of morbidity and mortality worldwide. The retention of apolipoprotein B (apoB)-containing lipoproteins within the arterial wall plays a major causal role in the initiation and progression of atherosclerosis. There is a single apoB molecule in all apoB-containing lipoproteins (intermediate-density lipoprotein, low-density lipoprotein (LDL), very low-density lipoprotein, lipoprotein(a) (Lp(a)), chylomicrons and chylomicron remnants). Therefore, quantitation of apoB directly estimates the number of atherogenic particles in plasma [1]. ApoB particle number has been shown to be a superior marker of cardiovascular risk than LDL-cholesterol (LDL-C) [2]. Guidelines state that apoB is the preferred measurement to refine the estimate of ASCVD risk [1].

Lp(a) is an LDL-like particle to which a molecule of apolipoprotein(a) has been disulfide bonded to apoB. Lp(a) is an established independent risk factor for ASCVD, independent of LDL-C. In addition to its cholesterol content, Lp(a) atherogenicity may be linked to the high content of oxidized phospholipids, which could promote inflammation, or to the structural similarity of apolipoprotein(a) to plasminogen and possibly effects on clotting. The median Lp(a) level in the white population is 19 nmol/L. Approximately 1.4 billion people worldwide have elevated Lp(a) concentrations (≥ 50 mg/dL; ≥125 nmol/L), which is the “rule in” cutoff for increased risk suggested by the European Atherosclerosis Society (EAS) consensus panel. The Lp(a) distribution is skewed to the right, particularly in the Caucasian population, in such a way that one person in 5 has Lp(a) levels over 6 times the median and one in 20 has Lp(a) levels over 10 times the median [3]. This implies that Lp(a)-mediated risk is distributed very unevenly in the population. In a recent Mendelian randomization study, Björnson et al. reported that the odds ratio of coronary heart disease (OR CHD) is 1.28 (95% confidence interval (CI): 1.24–1.33) per 50 nmol/L Lp(a)apoB and 1.04 (95% CI: 1.03–1.05) per 50 nmol/L LDLapoB. The atherogenicity of Lp(a) is approximately 7-fold greater than that of LDL on a per apoB particle basis [4]. We use the term atherogenicity to refer to the difference in CHD risk per unit difference in Lp(a) or LDL particle number.

In most individuals with normal Lp(a) and triglyceride (TG) levels, 90–95% of total apoB particles are LDL particles [2]. Therefore, LDL particles carry the majority of the overall atherogenic risk. However, in individuals with elevated Lp(a), this may not always be the case, and apoB may considerably underestimate risk. Furthermore, Arnold et al. recently reported a diminished or even lost association between apoB and incident CHD in subjects with increased Lp(a). On the other hand, LDL-C-related CHD estimates were greater in individuals with elevated Lp(a) levels. The authors were very intrigued by this unexplained, opposite pattern of association [5].

Thus, there is a need to understand the mechanisms of Lp(a), LDL-C- and apoB-related CHD risk. There is also a need to provide clinicians with a simple practical tool to address these complex and variable relationships. How can we understand a patient’s overall lipid-driven atherogenic risk? What proportion of this risk does apoB capture? What proportion of this risk do Lp(a) particles carry?

To answer these questions, we created a novel metric of atherogenic risk: risk-weighted apolipoprotein B.

## Methods

Simultaneous Lp(a) measurements in nmol/L and apoB measurements are needed.

ApoB levels are usually reported in mg/dL. ApoB can be easily converted from mg/dL to nmol/L by multiplying by 20 to obtain a molecular weight of 500 kDa [6].

ApoB and apolipoprotein(a) are present in Lp(a) at a molar ratio of 1/1 [3].

To obtain apoB other than Lp(a), Lp(a) in nmol/L is subtracted from apoB in nmol/L.

$$\eqalign{& {\rm{In}}\,{\rm{nmol}}/{\rm{L}}:{\bf{ApoB}}\>{\bf{other}}\>{\bf{than}}\>{\bf{ Lp}}\left( {\bf{a}} \right) \cr & \,\,\,\,\,\,\,\,\,\,\,\,\,\,\,\,\,\,\,\,\,\,\,\, = {\bf{apoB}} - {\bf{Lp}}\left( {\bf{a}} \right) \cr}$$

Mendelian randomization studies estimate that Lp(a) atherogenicity is approximately 7-fold greater than that of LDL on a per apoB particle basis [4]. Therefore, risk-weighted apoB is calculated as follows: in nmol/L

$$\eqalign{& {\bf{Risk}}\>{\bf{weighted}}\>{\bf{apoB}} \cr & \,\,\,\,\,\,\,\,\,\,\,\,\,\,\,\,\, = {\bf{apoB}}\> - \>{\bf{Lp}}\left( {\bf{a}} \right) + {\bf{Lp}}\left( {\bf{a}} \right)\>{\bf{\times}}\>7 \cr & \,\,\,\,\,\,\,\,\,\,\,\,\,\,\,\,\, = {\bf{apoB}} + {\bf{Lp}}\left( {\bf{a}} \right)\>{\bf{\times}}\>6 \cr}$$

$$\eqalign{& {\bf{Proportion}}\>{\bf{of}}\>{\bf{risk}}\>{\bf{captured}}\>{\bf{by}}\>{\bf{apoB}} \cr & \,\,\,\,\,\,\,\,\,\,\, = {\bf{apoB}}\>{\bf{divided}}\>{\bf{by}}\>{\bf{risk}}\>{\bf{weighted}}\>{\bf{apoB}} \cr}$$

$$\eqalign{& {\bf{Proportion}}\>{\bf{of}}\>{\bf{risk}}\>{\bf{carried}}\>{\bf{by}}\>{\bf{Lp}}\left( {\bf{a}} \right) \cr & \,\,\,\,\,\,\,\,\,\,\,\,\,\,\,\, = {\bf{Lp}}\left( {\bf{a}} \right)\> \times\>7\>\>{\bf{divided}}\>{\bf{by}}\>{\bf{risk}}\>{\bf{weighted}}\>{\bf{apoB}} \cr}$$

**Fig. 1:**
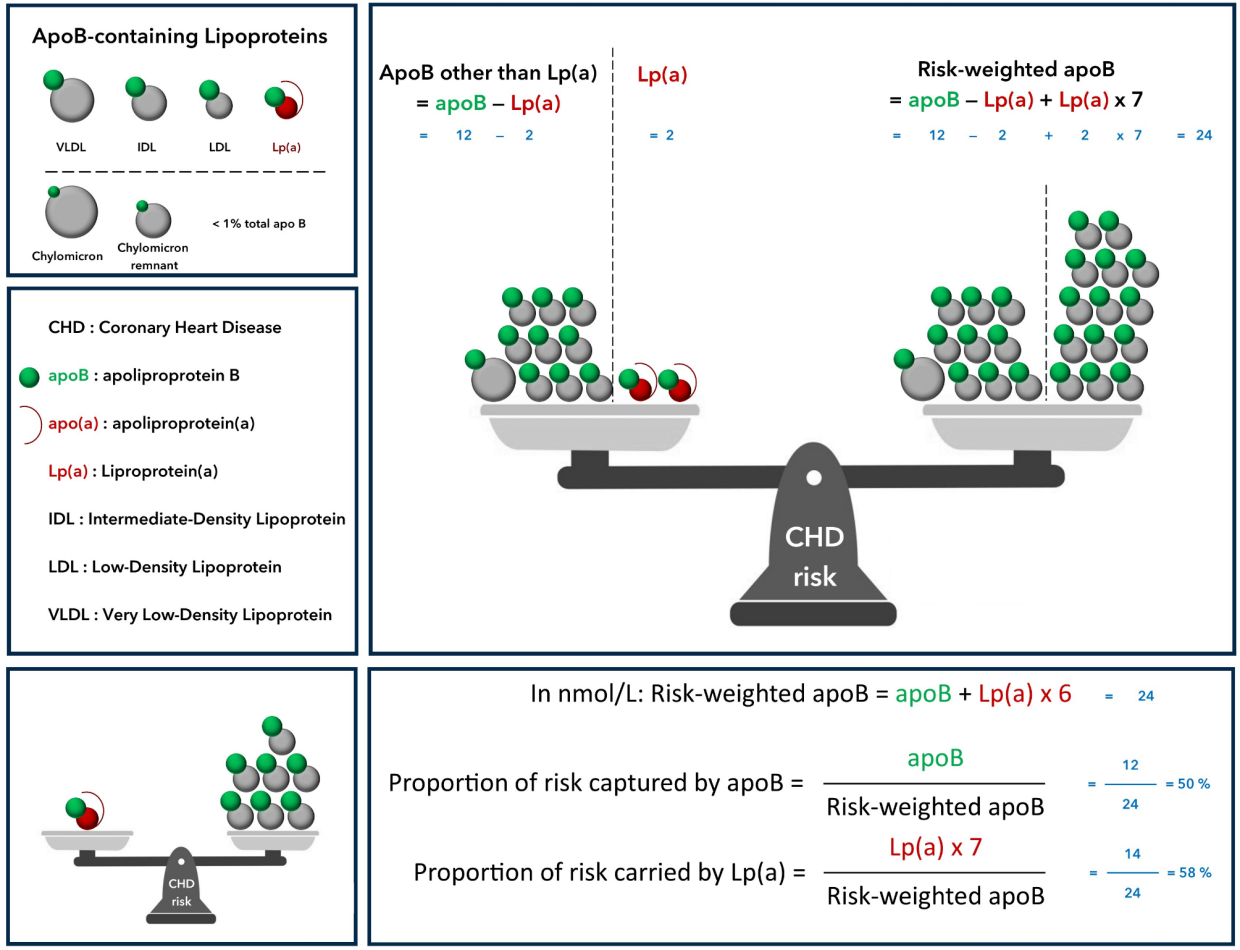
Lipoprotein(a) and risk-weighted apolipoprotein B: a novel metric for atherogenic risk

The results can then be converted back into mg/dL by dividing by 20 (Fig. 1).

## Results

The isoform size heterogeneity of Lp(a) does not support the use of a conversion factor between mass and molar units. However, to assess our metric in light of available evidence, in studies where only Lp(a) mass is reported, we use a conversion of 1 mg/dL = 2.5 nmol/L, which the EAS consensus panel describes as “the best guess” for a conversion factor [3].

### Risk-weighted apoB agrees with risk estimation from large epidemiological studies

On the basis of epidemiological data from 70 286 White individuals in the Copenhagen General Population Study with a median 7.4 years of follow-up, the risk of myocardial infarction approximately doubled at Lp(a) 250 nmol/L vs. the population median of 20 nmol/L. On the basis of epidemiological data from 415 274 White individuals from the UK biobank, the lifetime risk of major cardiovascular events approximately doubled at 250 nmol/L vs. the population median of 20 nmol/L [7]. In both datasets, a 230 nmol/L higher Lp(a) approximately doubled the risk [3]. With our tool, a patient with a 230 nmol/L higher Lp(a) has a risk-weighted apoB that is 1380 nmol/L higher (230 × 6). Atherogenicity increases by 4% per 50 nmol/L of risk-weighted apoB. Thus, for 1380 nmol/L higher risk-weighted apoB, our metric predicts a 110% risk increase (1380 × 4/50 = 110). This is within a very similar range to the doubling of the risk shown in these large epidemiological studies.

Arnold et al. published the largest study investigating the impact of Lp(a) level on LDL-C- or apoB-related risk of CHD in a prospective cohort of 68 748 CHD-free individuals followed for a median of 9.7 years for incident CHD events. They found, after multivariate adjustment, that an Lp(a) concentration of 25 mg/dL; 63 nmol/L vs. 3.5 mg/dL; 9 nmol/L was associated with a CHD risk increase of 26% [5]. For a 54 nmol/L higher Lp(a), our tool predicts an identical 26% risk increase. (54 × 6 × 4/50 = 26)

### Risk-weighted apoB agrees with risk estimation from other Mendelian randomization studies

Our tool is built on the genetic grounds of a very large (> 500 000 individuals) but unique dataset: the UK biobank [7]. Burgess et al. conducted a Mendelian randomization analysis on 48 333 participants of European descent (including 20 793 with CHD) using individual participant data from 5 studies other than the UK biobank. This analysis revealed that individuals with Lp(a) levels of 200 mg/dL; 500 nmol/L have an OR CHD of 3.30 (95% CI: 2.75–3.96), which is similar to the risk of CHD associated with heterozygous familial hypercholesterolemia [8]. For an individual with an Lp(a) of 500 nmol/L vs. the median of 20 nmol/L (480 nmol/L higher Lp(a)), our metric estimates an identical risk increase of 230% (480 × 6 × 4/50 = 230).

## Discussion

### Strengths

#### Compared with current practices, the use of risk-weighted apoB improves risk assessment and management in individuals with increased Lp(a)

First, LDL-C is the most widely used marker and target in the clinical care of ASCVD risk. This is problematic in individuals with elevated Lp(a). Owing to their overlapping densities, direct LDL-C measurement or LDL-C calculation with the Friedwald equation includes the cholesterol contained in Lp(a). “True LDL-C” is generally calculated via the Dahlen equation, which estimates that 30% of Lp(a) mass is cholesterol. However, the isoform size heterogeneity of Lp(a) does not support the use of mass units. Furthermore, the cholesterol content of Lp(a) is highly variable and, on average, much lower than 30% [9]. Thus, “true LDL-C” and LDL-C-driven risk are often underestimated in patients with high Lp(a). At present, we still do not know how to correct LDL-C for its Lp(a) cholesterol content. Risk-weighted apoB overcomes this long-lasting problem by capturing the global atherogenicity of a lipid panel without using LDL-C. Furthermore, the analytical performance of apoB measurements is superior to that of LDL-C measurements or calculations [10].

Second, although the association between Lp(a) and cardiovascular outcomes is linear from very low levels, for pragmatic reasons, current guidelines recommend the use of cutoffs for Lp(a)-mediated risk stratification [3]. However, selecting a threshold to determine when a relative risk becomes clinically meaningful is very difficult because the clinical impact largely depends on the patient’s absolute risk.

Arnold et al. investigated whether the correction of LDL-C for 30% Lp(a) mass cholesterol content affected the relationship between LDL-C and incident CHD. They chose the 90th percentile of their cohort (44 mg/dL; 110 nmol/L) to compare 2 groups: the high Lp(a) group and the low Lp(a) group [5].

In the high Lp(a) group, LDL-C was associated with higher risk estimates than in the low Lp(a) group. However, once LDL-C was corrected, the risk estimates in both groups were similar. In view of this, the authors showed that additional information on risk is included in Lp(a)-cholesterol when Lp(a) levels exceed 44 mg/dL; 110 nmol/L but not at the population level. However, 90% of the population was pooled in the low Lp(a) group (Lp(a) < 44 mg/dL; 110 nmol/L). In this pool, half of the individuals had Lp(a) levels under 8 mg/L; 20 nmol/L, and 75% had Lp(a) levels under 15 mg/dL; 38 nmol/L. Consequently, the risk estimates from this pool could be largely dominated by the predominance of subjects with (very) low Lp(a) levels. Although this analysis revealed that Lp(a)-cholesterol provides additional information on risk, when Lp(a) > 44 mg/dL; 110 nmol/L, it does not prove that Lp(a)-cholesterol adds information only above this threshold. Lp(a)-cholesterol could confer additional information below this cutoff. It is not stated if the authors tested several thresholds. Using thresholds to stratify Lp(a)-mediated risk is a limitation in current practice. When the authors analyzed Lp(a) as a sole biomarker, after multivariate adjustment, they reported that the association with incident CHD became significant above 11 mg/dL; 28 nmol/L (vs. reference range < 3.5 mg/dL; 9 nmol/L). This is in line with the risk increasing continuously with increasing Lp(a) concentration, already at levels below reported thresholds [3].

The EAS consensus panel suggests pragmatic Lp(a) cutoffs to ‘rule out’ (< 30 mg/dL; <75 nmol/L) or ‘rule-in’ (> 50 mg/dL;  > 125 nmol/L) risk. 30–50 mg/dL; 75–125 nmol/l is described as the gray zone [3]. However, Arnold et al. reported that in 60% of individuals with Lp(a) > 44 mg/dL; 110 nmol/L (which is in the EAS gray zone), apoB does not predict incident CHD risk. For the remaining 40%, the prediction is diminished [5]. Our instrument can predict risk at all levels of Lp(a) and apoB. In the EAS gray zone, our metric predicts an increased risk of 26% at 75 nmol/L and 50% at 125 nmol/L. Clinicians can consider this 26% relative risk increase, which seems particularly important when caring for patients with a high absolute risk.

With risk-weighted apoB, reasoning with Lp(a) thresholds is no longer necessary, which is a great improvement, as the association between Lp(a) and cardiovascular outcomes is linear from very low levels [3].

Third, multiple lines of evidence have shown that apoB is the best ASCVD risk marker at the population level [2]. However, it may not predict risk in individuals with high Lp(a), whereas we have shown with several large epidemiologic studies and several Mendelian randomization analyses that risk-weighted apoB does.

#### Risk-weighted apoB is a simple practical tool to help clinicians address Lp(a)-mediated risk

Clinicians can obtain an accurate and easy picture of a patient’s global lipid-driven atherogenic risk with just two measurements sampled at any time. Available immunoassays to measure apoB recognize both apoB48 and apoB100. However, fasting measurement is not necessary because even in the postprandial state, apoB48-containing chylomicrons generally represent less than 1% of circulating apoB-containing particles [1].

By using apoB as a common denominator, clinicians can compare the risk of patients with high Lp(a) levels to the risk of patients with high LDL-C levels.

Our formula can be used for risk assessment and reclassification at any level of apoB and Lp(a), with no need to use cutoff values. A patient with an apoB of 110 mg/dL and an Lp(a) of 19 nmol/L has a risk-weighted apoB of 116 mg/dL, the proportion of risk carried by Lp(a) is only 6%, and apoB captures 95% of risk. Clinicians can gauge the residual risk in patients on lipid-lowering therapy with well-controlled, low LDL-C but high Lp(a) levels. Let us consider a patient with an Lp(a) of 300 nmol/L and an apoB at the very high-risk target of 65 mg/dL [1]. The risk-weighted apoB is 155 mg/dL, the proportion of risk carried by Lp(a) is 68%, and apoB captures only 42% of the risk. This patient has a 135% greater risk than patients with apoB 65 mg/dL and Lp(a) 19 nmol/L. ((300 − 19)x6 × 4/50 = 135). These figures enable us to easily comprehend the considerable residual risk.

Furthermore, statin therapy has been shown to increase Lp(a) levels by 10–20% despite lowering LDL-C [11]. For most of the population with normal Lp(a) levels, this does not impact the overall benefit of statin therapy because of the small absolute increase in Lp(a). Indeed, Colantonio et al. reported that the association between Lp(a) and increased risk of CHD was not modified by statin use [12]. Nevertheless, in a few selected individuals, it could be interesting to assess the net benefit of treatment by comparing risk-weighted apoB before and during treatment. For patients with Lp(a) > 250 nmol/L, LDL-C-lowering therapy is recommended to mitigate ASCVD risk, even in primary prevention, regardless of absolute risk [3]. A patient with an apoB of 100 mg/dL and an Lp(a) of 250 nmol/L has a risk-weighted apoB of 175 mg/dL. The proportion of risk carried by Lp(a) is 50%. If during statin therapy, apoB is 75 mg/dL, and Lp(a) is 275 nmol/L (+ 10%), his risk-weighted apoB has only been reduced to 158 mg/dL. The proportion of risk carried by Lp(a) has risen to 61%.

#### Risk-weighted apoB explains the complex mechanisms of LDL-C- and apoB-related CHD risk in subjects with high Lp(a)

Arnold et al. reported that in subjects with Lp(a) above the 90th percentile of their cohort (44 mg/dL; 110 nmol/L), compared with the total population, the association of apoB with incident CHD was diminished when apoB was above 109 mg/dL (60th percentile) and was lost completely when apoB levels were below 109 mg/dL [5]. Risk-weighted apoB provides a mechanistic explanation for this phenomenon.

ApoB underestimates risk in patients with high Lp(a). The lower the apoB and the higher the Lp(a) are, the greater the underestimation. Thus, the underestimation is not uniform over the apoB quintiles. The underestimation is greatest in the reference range and decreases as apoB increases.

In this study, the first quintile of apoB was the reference (apoB < 80 mg/dL). We examined the proportion of risk captured by apoB in the second and top quintiles of apoB in both the low Lp(a) group (median Lp(a) 8 mg/dL; 20 nmol/L) and the high Lp(a) group (median Lp(a) 61 mg/dL; 153 nmol/L).

In the low Lp(a) group, for a patient with an apoB level of 135 mg/dL (top quintile) and an Lp(a) level of 8 mg/dL; 20 nmol/L, apoB accounts for 96% of the risk. For a patient with an apoB level of 87 mg/dL (second quintile) and an Lp(a) level of 8 mg/dL; 20 nmol/L, apoB accounts for 94% of the risk. The proportion of risk captured by apoB is very high. Thus, a statistically significant association is easily found.

In the high Lp(a) group, for a patient with an apoB of 135 mg/dL (top quintile) and an Lp(a) of 61 mg/dL;153 nmol/L, apoB accounts for 75% of the risk. For a patient with an apoB level of 87 mg/dL (second quintile) and an Lp(a) level of 61 mg/dL; 153 nmol/L, apoB only accounts for 65% of the risk. The risk estimations are so inaccurate that the statistical association is diminished or even lost.

Arnold et al. reported the opposite pattern of association with LDL-C. LDL-C-related CHD estimates were greater in subjects with higher Lp(a) masses [5]. This is also explained by risk-weighted apoB. In 2 subjects with the same LDL-C but different Lp(a) levels, the same mass of cholesterol is carried by a group of apoB particles that are, on average, more atherogenic in the subject with higher Lp(a). This finding is in line with the fact that beyond its cholesterol content, the greater atherogenicity of Lp(a) could be linked to the high content of oxidized phospholipids or to the structural similarity of apolipoprotein(a) to plasminogen. Furthermore, the presence of lysine in the kringle domains of apolipoprotein(a) contributes to the binding of Lp(a) to receptors on the endothelium. In addition, Lp(a) can be selectively retained in the arterial wall through the binding of apolipoprotein(a) to vascular extracellular matrix proteins [3].

### Limitations

Our tool is built on the genetic grounds of a very large (> 500 000 individuals) but unique dataset: the UK biobank [7]. We have shown that risk-weighted apoB agrees with risk prediction from other large Mendelian randomization studies. However, all these studies were conducted primarily in Caucasian populations and should be repeated in other ethnic groups.

Björnson et al. estimated that the atherogenicity of Lp(a) is 6.6 (95% CI: 5.1–8.8) greater than that of LDL on a per-particle basis [4]. We chose 7 as a multiplication factor because it corresponds to the OR CHD per 50 nmol/L (1.28 vs. 1.04, 28/4 = 7). Nevertheless, this is an approximation with a confidence interval and captures only the atherogenicity of Lp(a) with respect to CHD. Lp(a) has also been shown to be an independent risk factor for ischemic stroke, peripheral artery disease and aortic stenosis [3]. This multiplication factor and formula cannot be extrapolated to these other outcomes.

We considered that all apoB particles apart from Lp(a) had the same strength of association with CHD risk. Ference et al. showed that the CHD risk associated with variation in genes known to affect TG and, by extrapolation, triglyceride-rich lipoproteins (TRL) was similar to the risk associated with variation in genes affecting LDL for the same variation in apoB particle number. This strongly implied that the atherogenicity of TRL and LDL was broadly the same on a per apoB particle basis [13]. However, a more recent Mendelian randomization study suggested that TRL/remnant particles have approximately 2-fold greater atherogenicity than LDL particles on a per apoB particle basis [14]. This implies that our metric could slightly underestimate risk in individuals with elevated TG. Nonetheless, in subjects with normal TG levels, only 5–10% of total apoB particles are TRL/remnant particles. As plasma TG increases, this proportion can reach 15–20%, but even in severe hypertriglyceridemia (except for dysbetalipoproteinemia), LDL particles make up the great majority of apoB particles [2]. Furthermore, if we consider LDL particles as the benchmark, the 2-fold greater atherogenicity of TRL/remnant particles is considerably less than the 7-fold greater atherogenicity of Lp(a). Thus, the impact on risk-weighted apoB would be much smaller.

### Implications for future research

Our concept is a model for risk prediction that performs well in large epidemiological studies and Mendelian randomization studies from large different datasets. Our concept explains the complex associations among apoB, LDL-C and incident CHD in patients with elevated Lp(a).

Nevertheless, for further validation, randomized controlled trials testing the association between risk-weighted apoB and residual ASCVD risk in patients with high Lp(a) levels are necessary. Indeed, although our metric agrees with risk prediction from the Mendelian randomization study conducted by Burgess et al., the authors also showed that a 10 mg/dL genetically determined reduction in Lp(a) level was associated with a 5.8% CHD risk reduction, whereas a 14.5% CHD risk reduction was observed with a 10 mg/dL genetically determined reduction in LDL-C [8]. It is difficult to compare Lp(a) mass, which includes the mass of all the components of this particle (apoB, apolipoprotein(a), cholesterol) and LDL-C mass (which measures the cholesterol content of all low-density particles, including the cholesterol contained in Lp(a)). Thus, we cannot prove that our molar-based metric corroborates this finding.

Furthermore, post hoc analysis of the FOURIER trial (Further Cardiovascular Outcomes Research with PCSK9 Inhibition in Subjects with Elevated Risk) suggested that with a proprotein convertase subtilisin/kexin type 9 antibody, evolocumab, in secondary prevention, over 2.2 years, the relative risk reduction was 15% per 25 nmol/L Lp(a) decrease [15]. This implies that a 25 nmoL/L higher Lp(a) is associated with a 17% higher risk. (1/(1-0.15) = 1.17). In this situation, our metric predicts a 12% higher risk (25 × 6 × 4/50 = 12), which is lower. Nevertheless, this trial was not specifically designed to assess the Lp(a)-lowering effect of this agent. Risk-weighted apoB will need to be analyzed once we have the clinical outcome data from the ongoing phase III trials testing agents that specifically and potently lower Lp(a) concentrations by up to 90% by inhibiting apolipoprotein(a) synthesis.

## Conclusions

In subjects with high Lp(a), estimating cardiovascular risk with apoB considerably underestimates risk. The association between apoB and incident CHD is diminished or even lost. Risk-weighted apoB enables us to understand these phenomena, to gauge the risk in individuals with elevated Lp(a) and to quantify the proportion of overall lipid-driven atherogenicity carried by Lp(a).

## Data Availability

No datasets were generated or analysed during the current study.

## Abbreviations

ASCVD: Atherosclerotic cardiovascular disease
ApoB: Apolipoprotein B
LDL: Low-density lipoprotein
Lp(a): Lipoprotein(a)
LDL-C: Low-density lipoprotein cholesterol
EAS: European Atherosclerosis Society
OR CHD: Odds ratio for coronary heart disease
CI: Confidence interval
TG: Triglyceride
TRL: Triglyceride-rich lipoprotein
